# Impact of COVID-19 Diagnosis on Mortality in Patients with Ischemic Stroke Admitted during the 2020 Pandemic in Italy

**DOI:** 10.3390/jcm12144560

**Published:** 2023-07-08

**Authors:** Leonardo De Luca, Paola D’Errigo, Stefano Rosato, Gabriella Badoni, Barbara Giordani, Gian Francesco Mureddu, Andrea Tavilla, Fulvia Seccareccia, Giovanni Baglio

**Affiliations:** 1 Department of Cardio-Thoracic and Vascular Medicine and Surgery, Division of Cardiology, A.O. San Camillo-Forlanini, 00152 Rome, Italy; 2 Faculty of Medicine and Dentistry, UniCamillus-Saint Camillus International University of Health Sciences, 00131 Rome, Italy; 3 National Centre for Global Health, Italian National Institute of Health, 00161 Rome, Italy; paola.derrigo@iss.it (P.D.); stefano.rosato@iss.it (S.R.); gabriella.badoni@iss.it (G.B.); fulvia.seccareccia@iss.it (F.S.); 4 Italian National Agency for Regional Healthcare Services, 00187 Rome, Italy; barbara.giordani@agenas.it (B.G.); giovanni.baglio@agenas.it (G.B.); 5 Division of Cardiology, San Giovanni Addolorata Hospital, 00100 Rome, Italy; mureddu@gmail.com; 6 National Center for Disease Prevention and Health Promotion, Italian National Institute of Health, 00161 Rome, Italy; andrea.tavilla@agenas.it

**Keywords:** stroke, mortality, hospitalizations, national outbreak, COVID-19

## Abstract

Aims. The impact of the COVID-19 pandemic on the event rate of patients with ischemic stroke has been poorly investigated. We sought to evaluate the impact of the COVID-19 infection on mortality in patients with ischemic stroke admitted during the 2020 pandemic in Italy. Methods. We analyzed a nationwide, comprehensive, and universal administrative database of patients who were admitted for ischemic stroke during and after the national lockdown for the COVID-19 infection in 2020, and the equivalent periods over the previous 5 years in Italy. The 2020 observed hospitalization and mortality rates of stroke patients with and without COVID-19 infection were compared with the expected rates, in accordance with the trend of the previous 5 years. Results. During the period of observation, 300,890 hospitalizations for ischemic stroke occurred in Italy. In 2020, 41,302 stroke patients (1102 with concomitant COVID-19 infection) were admitted at 771 centers. The rate of admissions for ischemic stroke during the 2020 pandemic was markedly reduced compared with previous years (percentage change vs. 2015: −23.5). Based on the 5 year trend, the 2020 expected 30 day and 1 year mortality rates were 9.8% and 23.9%, respectively, and the observed incidence of death rates were 12.2% and 26.7%, respectively (both *p* < 0.001). After multiple corrections, higher rates of mortality were observed among patients admitted for stroke with a concomitant COVID-19 diagnosis. Conclusions. During the COVID-19 pandemic in 2020 in Italy, the rate of hospitalizations for ischemic stroke was dramatically reduced, although both the 30 day and 1 year mortality rates increased compared with the previous 5 year trend.

## 1. Introduction

The coronavirus disease of 2019 (COVID-19) has caused millions of deaths worldwide. In 2020, the pandemic mainly affected the north of Italy, where, especially during the national lockdown and the following few months, most of the confirmed cases of COVID-19-related fatal events occurred [1]. The COVID-19 pandemic has caused delays in emergency services and a profound reorganization of the healthcare system, causing a dramatic reduction in hospitalizations for acute and severe disorders, with a consequent increase in mortality and disability [1,2,3,4].

In patients with acute ischemic stroke, delays in care may have had a devastating impact on functional outcomes and survival, especially if access to rapid revascularization therapy was denied. The impact of the COVID-19 pandemic on the event rate of patients with ischemic stroke, especially during and immediately after the first peak of the epidemic [5,6], has been poorly investigated.

The aim of this study was to evaluate the impact of the COVID-19 infection on the trends of hospitalization and mortality rates in patients with ischemic stroke admitted during and after the national lockdown in 2020, in Italy, using a nationwide, comprehensive, and universal administrative database.

## 2. Methods

### 2.1. Study Design

This was a retrospective cohort study that enrolled patients admitted to all public and private hospitals in Italy for an ischemic stroke event and COVID-19 (11 March–3 May 2020) during the national lockdown, the post-lockdown period for COVID-19 in 2020 (4 May–31 December), and the equivalent periods (11 March–31 December) over the previous 5 years (pre-COVID-19 pandemic). We compared baseline characteristics, hospitalization rates, and 30 day and 1 year all-cause mortality rates between patients admitted for ischemic stroke during the lockdown and post-lockdown periods, for COVID-19 in 2020, and the prior 5 year equivalent periods. The Italian National Registry of Hospital Discharge Records (HDR), provided by the Italian Ministry of Health (MoH), and other administrative databases available in collaboration with the Italian National Program for Outcome Evaluation (PNE-AGENAS), were used as sources of data.

This study did not require specific approval from an ethical committee since it was conducted as part of public health research, which is included in the institutional mandate of the Italian National Institute of Health.

### 2.2. Study Population

All HDR of patients aged 35 to 100 years, who were resident in Italy, admitted during the study period, and who had reported a diagnosis of ischemic stroke were selected. For the purposes of this study, ischemic stroke patients were defined as patients with ICD 9 CM codes 433.x1, 434.x1, and 436 as part of their primary diagnosis [7].

Admissions with a diagnosis of hemorrhagic stroke, transient ischemic attack (ICD9 CM codes 430, 431, 432), psychic disorders (ICD9 CM codes 290–319), malignant neoplasm (ICD9 CM codes 140.0–208.9), childbirth, or other obstetric specialties (ICD9 CM codes 72–75 or DRG 370–384) were excluded from the analysis. Patients transferred from another hospital, and patients who had been diagnosed with a stroke within the previous five years, were also excluded.

In accordance with the Italian MoH documents released in 2020 for COVID-19 identification, ischemic stroke patients with a concomitant definite or suspected diagnosis of COVID-19 were defined as stroke cases with at least one of the following ICD 9 CM codes: 078.89 other specified diseases due to viruses (MoH first guidelines—20 March 2020); 043 COVID-19 disease; 480.4 COVID-19 Pneumonia; 518.9 COVID-19 Acute respiratory distress syndrome (ARDS); 519.7 COVID-19 Other respiratory infections (MoH Decree—28 October 2020); 079.82 SARS-associated coronavirus; 480.3 Pneumonia due to SARS-associated coronavirus (ICD-9-CM codes for SARS); and codes identifying exposure, isolation, anamnesis, observation (‘V01.85’, ‘V01.79’, ‘V71.83’, ’V07.0’, ‘V71.84’, ‘V07.00’, ‘V12.04’, ‘V01.82’), or pneumonia in other infectious diseases (484.8) [7].

Details on patient risk factors and comorbidities, in accordance with the ICD9-CM codes reported in Supplementary Table S1, were obtained either from the index admission or previous hospitalizations over the past 5 years.

To assess the impact of COVID-19 on mortality in different areas of the country, three macro-regions of Italy were selected: Northern (Lombardia, Piemonte, Valle d’Aosta, Veneto, Friuli Venezia-Giulia, Trentino Alto-Adige, Liguria, and Emilia-Romagna; accounting for a total of 13,480,648 inhabitants in 2020), Central (Lazio, Toscana, Umbria, and Marche; 5,719,084 inhabitants), and Southern (Abruzzo, Molise, Puglia, Basilicata, Campania, Calabria, Sicilia, and Sardegna; 9,850,364 inhabitants).

The 30 day and 1 year all-cause mortality rates indicated the main adverse outcomes.

### 2.3. Statistical Analysis

Prevalence of risk factors and comorbidities were presented as counts and percentages, and age and length of stay (LOS) were expressed as the mean ± standard deviation.

*T*-Test, Chi-square, or Fisher exact tests were used to compare frequencies between pre-lockdown, lockdown, and post-lockdown periods and between COVID-19 and non- COVID-19 patients in the 2020 stroke cohort, as appropriate.

The number of expected stroke events and the rates of the comorbidities and outcomes in 2020 were estimated using a linear regression model, using the number of stroke events and the rates of the comorbidities and outcomes in the prior 5-year equivalent periods as predictors. The number of actual and expected events in the 2020 study period were compared using the Poisson test. The observed and expected rates of both comorbidities and outcomes were compared using the log-normal distribution property of the rate ratio (H0: observed rate/expected rate = 1).

The normal distribution of continuous parameters was tested with the Kolmogorov–Smirnov test. Variables with a skewed distribution were compared with the use of Wilcoxon rank sum tests.

To provide adjusted outcome data, age, gender, thrombolysis administered ≤48 h from hospital admission, department of admission (stroke unit or not), and patients’ risk factors and comorbidities were included in the multivariate models as potential confounding factors. Stepwise procedures were used to identify independent associations with each of the considered outcomes. The 30 day mortality rate was analyzed using a logistic model, and the Cox model was used to analyze the 1 year mortality rate. Since some chronic comorbidities recorded in the index hospitalization exhibited a paradoxical protective effect [8], the same comorbidities recorded in previous hospitalizations were also entered into the models.

All assumptions of statistical methods were explicitly checked. Statistical analyses were performed using SAS 9.4 (Cary, NC, USA).

## 3. Results

During the study period, 300,890 hospitalizations for ischemic stroke occurred in Italy. In 2020, 41,302 patients with ischemic stroke were admitted at 771 centers: 6586 during almost 8 weeks of national outbreak and 34.716 after the lockdown. Among these patients, 1102 had a COVID-19 diagnosis. In 2020, patients admitted for ischemic stroke and COVID-19 often had a history of heart failure and a longer LOS; it was less common for these patients to have a vascular and chronic kidney disease at admission, to be admitted to a stroke unit, or to receive a thrombolysis within 48 h from hospital admission, compared with those without a COVID-19 diagnosis (Supplementary Table S2).

**Figure 1:**
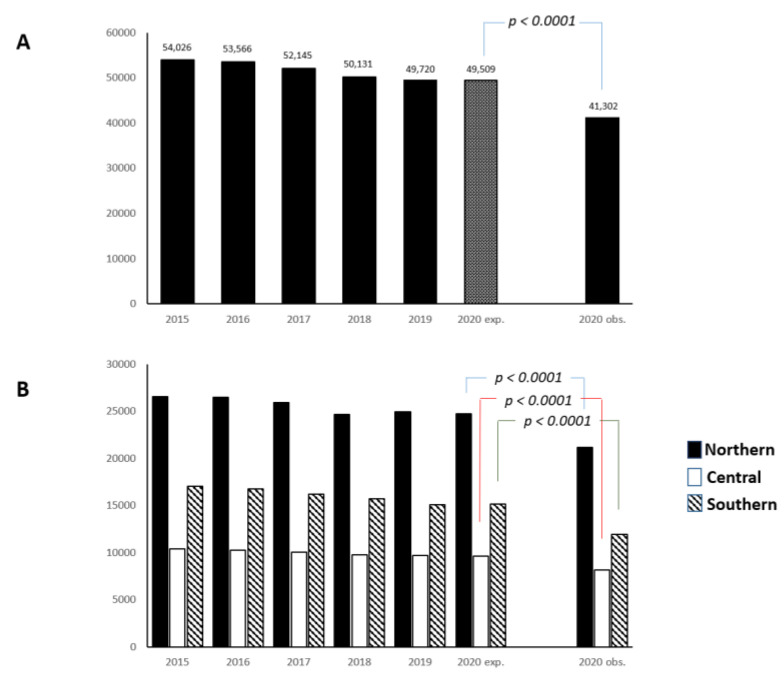
Expected (exp.) and observed (obs.) incidence of ischemic stroke admission during 2020 (11 March–31 December), over the equivalent periods in the previous 5 years in Italy (**panel A**), and by geographic region (**panel B**).

Compared with the previous 5 years, the rate of admissions for ischemic stroke in 2020 was markedly reduced (from 54,026 in 2015 to 41,302 in 2020; percentage change −23.5). Considering the 5 year trend, the observed number of stroke admissions in 2020 was significantly reduced as compared with the expected number (41,302 vs. 49,509; *p* < 0.0001) (Figure 1A). The reduced rate in the number of stroke admissions in 2020, as compared with the expected rate based on trends, was consistent in Northern, Central, and Southern Italy (all *p*-values < 0.0001) (Figure 1B).

Demographic and clinical characteristic trends of patients admitted for stroke during each year of observation are depicted in Supplementary Table S3. Patients admitted for stroke during 2020 (lockdown and post-lockdown periods) presented several differences in terms of baseline clinical characteristics compared with those hospitalized during the previous 5 years of observation. For instance, mean age, number of females, patients with diabetes mellitus, anemia, cerebrovascular diseases, chronic kidney disease, LOS, history of myocardial infarction, and heart failure were reduced, whereas patients treated with thrombolysis < 48 h from admission, and those admitted in a stroke unit, were significantly increased in 2020 compared with 2015–2019 (Table 1).

### Mortality Trends

According to the 5 year trend, the 2020 expected rate with regard to the 30 day all-cause mortality rate was 9.8%, whereas the observed incidence of death rate was 12.2% (*p* < 0.001). Excluding patients with a COVID-19 diagnosis, the observed incidence regarding the 30 day mortality rate was 11.9% (*p* < 0.0001 compared with the expected trend rate) (Supplementary Figure S1).

**Figure 2:**
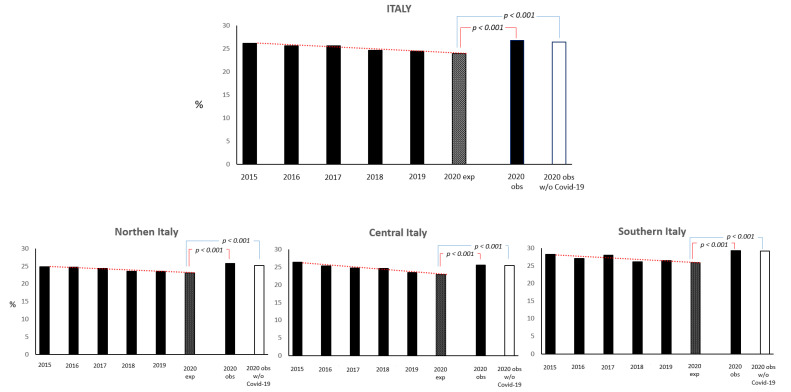
Expected (exp.) and observed (obs.) mortality rate at 1 year (in all ischemic stroke patients and in those without COVID-19 infection) during 2020 (11 March–31 December), over the equivalent periods in the previous 5 years in Italy, and by geographic region.

Accordingly, the 2020 expected rate regarding the 1 year all-cause mortality rate was 23.9%, whereas the observed incidence of death rate was 26.7% (*p* < 0.001); after excluding patients with COVID-19, the observed incidence of mortality at 1 year was 26.4% (*p* < 0.0001 compared with the expected trend rate) (Figure 2). The difference in the observed rates, regarding the 30 day and 1 year mortality rates of patients admitted for ischemic stroke during 2020, with and without a COVID-19 diagnosis, was consistent in all three areas of Italy (Supplementary Figures S1 and S2). Comparing the fatality rates of stroke patients without a COVID-19 infection during the period of observation, the difference between the expected and observed 30 day and 1 year mortality rates was particularly evident for those not hospitalized in a stroke unit (16.0 vs. 19.3%, percentage change +17.1; and 33.6% vs. 36.7%, percentage change +8.4, respectively) (Supplementary Figure S2).

**Figure 3:**
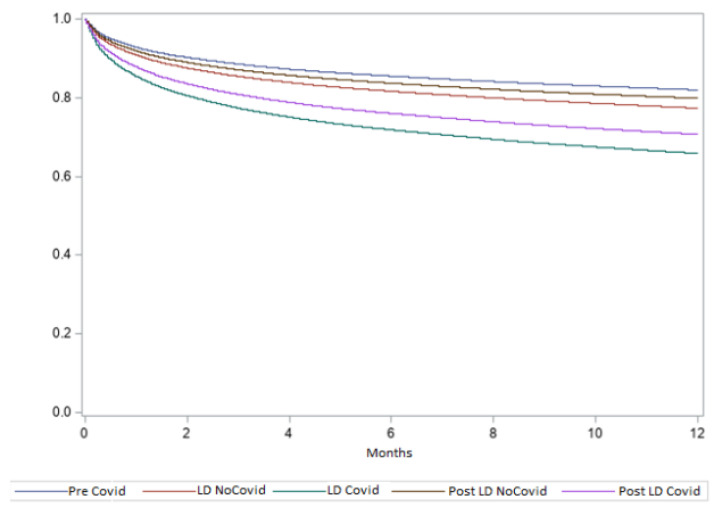
Cox-adjusted survival curves regarding the 1 year mortality rate of stroke patients, with or without a COVID-19 diagnosis, admitted in Italy during the pre-COVID-19 (11 March–31 December of 2015–2019), lockdown (*LD*; 11 March–3 May 2020), and post-lockdown (Post LD; 4 May–31 December 2020) periods.

After multiple corrections, a higher rate of mortality at 1 year was observed among patients admitted for ischemic stroke with a concomitant COVID-19 infection during the 2020 lockdown and post-lockdown periods (Figure 3). Moreover, the presence of a COVID-19 diagnosis during the lockdown and post-lockdown periods resulted in the most powerful independent predictors of all-cause mortality at 30 days (adjusted odds ratio (OR) 3.68; 95% confidence intervals (CI) 2.75–4.88 and adjusted OR 2.49; 95% CI: 2.08–2.97, respectively; both *p* < 0.0001) (Supplementary Table S4) and 1 year (adjusted hazard ratio (HR) 2.11; 95% CI: 1.76–2.52 and adjusted HR 1.75; 95% CI: 1.56–1.96, respectively; both *p* < 0.0001) among stroke patients (Table 2).

## 4. Discussion

The major findings of this analysis, which used a nationwide, universal administrative database of patients admitted for ischemic stroke during the COVID-19 pandemic in 2020 in Italy, are as follows: 1. a significant reduction in 2020 ischemic stroke hospitalization rates and a marked increase in expected and observed mortality rates was documented, as compared with the prior 5 year trend; 2. after multiple adjustments, the highest 1 year mortality rate was observed in stroke patients with COVID-19 who were admitted during and after the lockdown in 2020, compared with those without COVID-19 hospitalized in 2020 and in the previous years; 3. among patients without COVID-19, the increased mortality rate was particularly evident for those not admitted to stroke units.

We found a significant 23% relative reduction in the number of patients hospitalized for ischemic stroke during the COVID-19 pandemic. Accordingly, we previously documented substantial reductions in hospitalization rates for other acute conditions such as acute myocardial infarction during the COVID-19 pandemic, even if we used different patient cohorts and periods of observation [4]. The low rate of hospitalizations for stroke is consistent with a survey conducted during national lockdown in 93 Italian Stroke units [8]. Other previous studies reported a reduction in hospitalization rates for stroke during COVID-19 pandemic, ranging from 18% to 48% [5,6,9,10,11,12,13]. These different rates may be explained, in part, by different health systems and strategies for managing the COVID-19 pandemic; however, they are mainly explained by the fact that other reports retrospectively analyzed data of all stroke types (ischemic and hemorrhagic, sometimes also including transient ischemic attacks) in a specific, short period of time, and these data were collected from specialized stroke units only, or a few centers or regional areas where COVID-19 had significantly spread [5,6,9,10,11,12,13]. Indeed, a recent analysis of two large French regions, differently affected by the COVID-19 crisis, demonstrated a significant reduction in stroke admission rates in the most severely affected region only, and no changes were observed in the least affected region of the 2020 pandemic, as compared with the same month in the previous years [9]. To our knowledge, this is the first study that considered a widespread nationwide database of ischemic strokes only, in addition to a long period of COVID-19 infection. Therefore, our study included nonspecialized public and private hospitals, with or without stroke units, and it considered the first year of the COVID-19 pandemic, including the peak of infection which occurred during lockdown, and subsequent periods.

The reasons for the dramatic reduction in hospitalization rates for strokes, as documented in our analysis, may be related to the fact that subjects with suspected acute stroke may have been afraid of exposure to COVID-19 in hospitals [11,14], or they had difficulties identifying symptoms early, with health assistance, or with transportation during the pandemic.

Some studies observed a significant reduction in the administration of acute revascularization therapies in cases of acute stroke, such as intravenous thrombolysis, during the COVID-19 pandemic [10,11,15,16]. Nevertheless, an analysis of the Big Data Observatory Platform for Stroke of China [10] found a 27% reduction in the absolute number of acute pharmacological revascularizations in 2020; this was due to the relative reduction in stroke admissions. However, thrombolysis rates increased by 9.4% compared with the previous year; this trend is in accordance with the US report by the Get With The Guidelines Registry, published few years before [17]. We also observed an increase in the relative rate of thrombolysis administered within 24 h from admission, a gauge included in our HDR, together with a growth in the number of stroke patients hospitalized in stroke units compared with previous years. This trend suggests a global improvement in stroke management and greater adherence to international guidelines and recommendations which extends beyond the COVID-19 epidemic. Indeed, in our series, as in previous studies [9,18], the rate of thrombolysis, and stroke units’ admissions, was significantly reduced among those with COVID-19 compared with stroke patients without a COVID-19 diagnosis.

We observed increased short- and long-term mortality rates in patients with strokes who were hospitalized in Italy during the 2020 COVID-19 pandemic compared with the previous 5 year trend. Wide-ranging authority policies, scarce medical resources, avoidance of healthcare settings due to the population’s fear of contracting the infection, and the reorganization of healthcare system leading to hospitalization of patients with stroke in non-specialized units, may comprise reasons for the high fatality rate detected during the pandemic [19,20]. In this regard, a significantly higher rate of death among stroke patients who were not admitted into stroke units in 2020, compared with previous years, was noted in our series. Notably, the highest adjusted mortality rate was documented in stroke patients with a concomitant COVID-19 infection, who were admitted during the lockdown; this period comprised the widest spread of COVID-19 and the highest rate of mortality related to the infection [21]. This finding suggests that COVID-19 may have impacted on prognosis of patients with ischemic stroke. Indeed, in our analysis, a concomitant COVID-19 infection was associated with a higher mortality rate after the lockdown compared with patients without a COVID-19 diagnosis; this was the case for patients hospitalized in Italy during 2020 and in the prior 5 years. Accordingly, in a population-based study analyzing cerebrovascular-related excess mortality rates during the COVID-19 pandemic in 40 US States, excess stroke mortalities were observed during the first wave of the pandemic [20]. Although previous reports have suggested that experiencing a stroke during a COVID-19 infection could be related to hypercoagulability and endotheliopathy, which can cause a thrombotic microangiopathy or a paradoxical embolism [22,23,24,25], the mechanisms underlying this excess mortality rate requires further investigation.

### Limitations

There are several limitations when using an administrative health claims database. First, there is a lack of specific clinical information, which may have affected the accuracy of the diagnosis, severity, and risk stratification of stroke. In addition, access times to hospital and revascularization procedures were not analyzed, and neither were disability and quality of life procedures. Moreover, changes in stroke unit admissions can depend on the number of available beds, which may have varied during the COVID-19 pandemic, thus limiting the interpretation of these data. Another limitation concerns the deficiencies in the ICD-9 CM code descriptions when providing information on drug use, parameters derived from clinical tests (e.g., blood tests), as well as comprehensive data on in-hospital complications and causes of death. In this regard, it was not possible to completely rule out variables associated with COVID-19 diagnoses that may have produced an uncontrolled bias in terms of their association with mortality. Finally, potential misclassifications, coding errors, or under-detection-related bias may have occurred, especially for comorbidities.

## 5. Conclusions

During the COVID-19 pandemic in 2020 in Italy, the rate of hospitalizations for ischemic stroke was dramatically reduced, whereas both 30 day and 1 year mortality rates markedly increased compared with the previous 5 year trend. After multiple adjustments, the highest 1 year mortality rate was observed in ischemic stroke patients with COVID-19 who were admitted during and after the lockdown in 2020, compared with those without a COVID-19 diagnosis who were hospitalized between 2015 and 2020.

These findings suggest that the COVID-19 pandemic has substantially constrained patient referrals and access to the emergency health care system. Moreover, a concomitant COVID-19 infection has further worsened the clinical conditions of ischemic stroke patients, with a significant impact on case fatality. Health authorities should be apprised of these findings in order to implement proper public health strategies, such as building confidence in health services through the community, and strengthening logistics and the supply of resources, in the context of a possible future pandemic crisis.

## Figures and Tables

**Table 1:** Baseline characteristics of patients with ischemic stroke admitted in 2015–2019 (11 March–31 December), during the 2020 lockdown (11 March–3 May 2020), and in 2020 post-lockdown (4 May–31 December 2020) in Italy.

	2015–2019 (N = 259,588)	2020 Lockdown (N = 6586)	2020 Post-Lockdown (N = 34,716)	*p*-Value
Gender (females), n (%)	128,630 (49.6)	3214 (48.8)	16710 (48.1)	<0.001
Age (years), mean ± SD	75.9 ± 12.3	75.7 ± 12.2	75.5 ± 12.4	<0.001
Malignant neoplasms, n (%)	20,917 (8.1)	525 (8.0)	2781 (8.0)	0.93
Diabetes mellitus, n (%)	27,114 (10.4)	545 (8.3)	3034 (8.7)	<0.001
Dyslipidemias, n (%)	9797 (3.8)	221 (3.4)	1134 (3.3)	<0.001
Obesity, n (%)	3892 (1.5)	87 (1.3)	500 (1.4)	0.36
Obesity (ind. adm.), n (%)	2648 (1.0)	91 (1.4)	521 (1.5)	<0.001
Anemia, n (%)	12,402 (4.8)	280 (4.3)	1467 (4.2)	<0.001
Anemia (ind. adm.), n (%)	6217 (2.4)	157 (2.4)	864 (2.5)	0.56
Blood clotting defects, n (%)	534 (0.2)	14 (0.2)	55 (0.2)	0.18
Blood clotting defects (ind. adm.), n (%)	288 (0.1)	7 (0.1)	41 (0.1)	0.92
Other hematological diseases, n (%)	1510 (0.6)	34 (0.5)	207 (0.6)	0.73
Other hematological diseases (ind. adm.), n (%)	1197 (0.5)	32 (0.5)	195 (0.6)	0.04
Hypertension, n (%)	50,247 (19.4)	1085 (16.5)	5345 (15.4)	<0.001
Previous myocardial infarction, n (%)	12,684 (4.9)	250 (3.8)	1477 (4.3)	<0.001
Heart failure, n (%)	21,903 (8.4)	469 (7.1)	2489 (7.2)	<0.001
Chronic coronary syndromes, n (%)	23,516 (9.1)	510 (7.7)	2599 (7.5)	<0.001
Rheumatic heart disease, n (%)	3266 (1.3)	63 (1.0)	343 (1.0)	<0.001
Rheumatic heart disease (ind. adm.), n (%)	1921 (0.7)	58 (0.9)	363 (1.0)	<0.001
Cardiomyopathy, n (%)	3769 (1.5)	83 (1.3)	427 (1.2)	0.002
Cardiomyopathy (ind. adm.), n (%)	1247 (0.5)	32 (0.5)	163 (0.5)	0.99
Endocarditis and acute myocarditis, n (%)	352 (0.1)	9 (0.1)	43 (0.1)	0.85
Arrhythmias, n (%)	32,296 (12.4)	704 (10.7)	3567 (10.3)	<0.001
Other chronic heart conditions, n (%)	6355 (2.4)	163 (2.5)	814 (2.3)	0.49
Other chronic heart conditions (ind.adm.), n (%)	6591 (2.5)	189 (2.9)	1068 (3.1)	<0.001
Vascular disease, n (%)	12,706 (4.9)	288 (4.4)	1404 (4.0)	<0.001
Vascular disease (ind. adm.), n (%)	8747 (3.4)	245 (3.7)	1390 (4.0)	<0.001
Chronic obstructive pulmonary disease, n (%)	14,382 (5.5)	283 (4.3)	1410 (4.1)	<0.001
Chronic kidney disease, n (%)	14,042 (5.4)	341 (5.2)	1556 (4.5)	<0.001
Chronic kidney diseases (ind. adm.), n (%)	11,582 (4.5)	286 (4.3)	1420 (4.1)	0.006
Other chronic disease (liver, pancreas, intestine), n (%)	4401 (1.7)	70 (1.1)	459 (1.3)	<0.001
Other chronic disease (liver, pancreas, intestine) (ind. adm.), n (%)	2152 (0.8)	31 (0.5)	221 (0.6)	<0.001
Previous coronary revascularization, n (%)	12,399 (4.8)	314 (4.8)	1581 (4.6)	0.19
Previous coronary revascularization (ind. Adm.), n (%)	5699 (2.2)	151 (2.3)	676 (1.9)	0.009
Previous vascular surgery, n (%)	11,106 (4.3)	291 (4.4)	1398 (4.0)	0.07
Thrombolysis < 48 h, n (%)	25,872 (10.0)	791 (12.0)	4228 (12.2)	<0.001
Stroke unit, n (%)	160,928 (62.0)	4643 (70.5)	24105 (69.4)	<0.001
LOS (mean ± SD)	10.3 ± 9.4	9.4 ± 8.7	9.6 ± 7.7	<0.001
COVID-19 diagnosis	0	274 (4.2)	828 (2.4)	<0.001

Abbreviations: COVID-19: coronavirus disease 2019; ind. adm.: index admission; LOS: length of stay.

**Table 2:** Cox regression model for the 12 month mortality rate.

	HR	95% CI		*p*-Value
Gender, females	1.07	1.06	1.09	<0.0001
Age, years	1.09	1.08	1.09	<0.0001
Pre-COVID-19	Ref			
Lockdown—No COVID-19	1.29	1.23	1.35	<0.0001
Lockdown—COVID-19	2.11	1.76	2.52	<0.0001
Post-Lockdown—No COVID-19	1.14	1.11	1.16	<0.0001
Post-Lockdown—COVID-19	1.75	1.56	1.96	<0.0001
Malignant neoplasms	1.26	1.23	1.29	<0.0001
Diabetes mellitus	1.23	1.20	1.26	<0.0001
Dyslipidemia	0.81	0.78	0.85	<0.0001
Obesity	1.11	1.05	1.17	0.0002
Anemia	1.18	1.15	1.21	<0.0001
Anemia (ind. adm.)	1.12	1.08	1.17	<0.0001
Blood clotting defects	1.18	1.04	1.33	0.008
Blood clotting defects (ind. adm.)	1.62	1.29	2.05	<0.0001
Other hematological diseases	1.29	1.20	1.39	<0.0001
Hypertension	0.97	0.95	0.99	0.003
Previous myocardial infarction	1.10	1.06	1.14	<0.0001
Heart failure	1.37	1.34	1.40	<0.0001
Rheumatic heart disease	1.08	1.02	1.13	0.004
Rheumatic heart disease (ind. adm.)	0.76	0.70	0.83	<0.0001
Cardiomyopathy	1.23	1.17	1.29	<0.0001
Endocarditis and acute myocarditis	1.26	1.07	1.48	0.006
Other chronic heart conditions	1.09	1.05	1.14	<0.0001
Other chronic heart conditions (ind.adm.)	0.81	0.76	0.86	<0.0001
Arrhythmias	1.22	1.20	1.25	<0.0001
Vascular disease	1.13	1.09	1.17	<0.0001
Vascular disease (ind. adm.)	0.77	0.74	0.81	<0.0001
Chronic obstructive pulmonary disease	1.20	1.17	1.23	<0.0001
Chronic kidney disease	1.21	1.18	1.24	<0.0001
Chronic kidney diseases (ind. adm.)	1.08	1.05	1.11	<0.0001
Other chronic disease (liver, pancreas, intestine)	1.20	1.14	1.26	<0.0001
Other chronic disease (liver, pancreas, intestine) (ind. adm.)	1.22	1.12	1.32	<0.0001
Previous coronary revascularization	0.86	0.83	0.90	<0.0001
Previous vascular surgery	1.31	1.27	1.36	<0.0001
Thrombolysis < 48 h	0.93	0.91	0.96	<0.0001
Stroke unit admission	0.76	0.75	0.78	<0.0001

Abbreviations: COVID-19: coronavirus disease 2019; ind. adm.: index admission.

## Data Availability

The data presented in this study are available on request from the corresponding author.

## Supplementary Materials

The following supporting information can be downloaded at: https://www.mdpi.com/article/10.3390/jcm12144560/s1, Figure S1: Expected (exp.) and observed (obs.) mortality rate at 30 days (in all ischemic stroke patients and in those without COVID-19 infection) during the 2020 and over the equivalent periods in the previous 5 years in Italy and by geographic regions; Figure S2: Mortality rate at 30 days (panel A) and at 1 year (panel B) among patients without Covid-19 infection admitted in neurology and non-neurology units during the 2020 and over the equivalent periods in the previous 5 years in Italy. Table S1: ICD9-CM codes used to retrieve information on risk factors and comorbidities; Table S2: Baseline characteristics of patients without and with COVID-19 infection; Table S3: Baseline characteristics of the enlisted population by year; Table S4: Logistic regression model for 30-day mortality.
