# Supplementary material for: Impact of COVID-19 Diagnosis on Mortality in Patients with Ischemic Stroke Admitted during the 2020 Pandemic in Italy
Source: J Clin Med. 2023 Jul 8;12(14):4560. doi: 10.3390/jcm12144560 (PMC10380878; doi:10.3390/jcm12144560)

SUPPLEMENTARY TABLE S1. ICD9-CM codes used to retrieve information on risk factors and comorbidities

	ICD9 Code	
	**Index admission**	**Previous admission**
Hypertension		401-405
Diabetes		250.0-250.9
Heart failure		428
Ill-defined descriptions and complications of heart disease		429
Cerbrovascular disease		430-432, 433, 434, 436, 437, 438
Vascular disease	440-448 (except 441.1, 441.3, 441.5, 441.6, 444), 557.1	440-448, 557
Chronic coronary syndromes		411, 413, 414
Arrhythmias		426, 427
Anemia	280-284, 285 (except 285.1)	280-284, 285 (except 285.1)
Blood clotting defects	286	286
Other hematological diseases	287-289	287-289
Cardiomyopathy	425	425
Rheumatic heart disease	393-398	391, 393-398
Endocarditis and acute myocarditis		421, 422
Other chronic heart conditions	745, V15.1, V42.2, V43.2, V43.3, V45.0	745, V15.1, V42.2, V43.2, V43.3, V45.0
Chronic kidney diseases	582, 583, 585-588	582, 583, 585-588
Chronic disease (liver, pancreas, intestine)	571, 572, 577.1-577.9, 555, 556	571, 572, 577.1-577.9, 555, 556
Obesity	278.0	278.0
Chronic obstructive pulmonary disease		491, 492, 494, 496
Malignant neoplasms	V10	140.0–208.9, V10
Previous vascular surgery		38-39.5 (except: 38.01, 38.02, 38.5, 38.11, 38.12, 38.31, 38.32)
Previous cerebral revascularization		00.61, 00.62, 38.01, 38.02, 38.11, 38.12, 38.31, 38.32
Other previous cardiac surgery than CABG		35, 37.0, 37.1, 37.3, 37.4, 37.5, 37.6, 37.9
Disorders of lipid metabolism		272
Previous AMI	412	410, 412
Previous coronary revascularization	V45.81, V45.82	V45.81, V45.82,36.0, 36.1, 00.66
Thrombolysis	9910	

SUPPLEMENTARY TABLE S2. Baseline characteristics of patients without and with Covid-19 infection.

	No COVID-19 (N=40200)	COVID-19 (N=1102)	p value
Gender (females), n (%)	19380 (48.2)	544 (49.4)	0.45
Age (years), mean±SD	75.5±12.4	75.8±12.7	0.38
Malignant neoplasms, n (%)	3225 (8.0)	81 (7.4)	0.42
Diabetes mellitus, n (%)	3499 (8.7)	80 (7.3)	0.09
Dislipidemias, n (%)	1329 (3.3)	26 (2.4)	0.08
Obesity, n (%)	578 (1.4)	9 (0.8)	0.09
Obesity (ind. adm.), n (%)	599 (1.5)	13 (1.2)	0.40
Anemia, n (%)	1688 (4.2)	59 (5.4)	0.06
Anemia (ind. adm.), n (%)	995 (2.5)	26 (2.4)	0.81
Blood clotting defects, n (%)	65 (0.2)	4 (0.4)	0.11
Blood clotting defects (ind. adm.), n (%)	48 (0.1)	0	0.25
Other hematological diseases, n (%)	234 (0.6)	7 (0.6)	0.82
Other hematological diseases (ind. adm.), n (%)	221 (0.5)	6 (0.5)	0.98
Hypertension, n (%)	6271 (15.6)	159 (14.4)	0.29
Previous myocardial infarction, n (%)	1675 (4.2)	52 (4.7)	0.37
Heart failure, n (%)	2856 (7.1)	102 (9.3)	0.006
Chronic coronary syndromes, n (%)	3036 (7.6)	73 (6.6)	0.25
Rheumatic heart disease, n (%)	397 (1.0)	9 (0.8)	0.57
Rheumatic heart disease (ind. adm.), n (%)	413 (1.0)	8 (0.7)	0.33
Cardiomyopathy, n (%)	489 (1.2)	21 (1.9)	0.04
Cardiomyopathy (ind. adm.), n (%)	192 (0.5)	3 (0.3)	0.33
Endocarditis and acute myocarditis, n (%)	49 (0.1)	3 (0.3)	0.17
Arrhythmias, n (%)	4134 (10.3)	137 (12.4)	0.02
Other chronic heart conditions, n (%)	956 (2.4)	21 (1.9)	0.31
Other chronic heart conditions (ind.adm.), n (%)	1238 (3.1)	19 (1.7)	0.01
Vascular disease, n (%)	1654 (4.1)	38 (3.4)	0.27
Vascular disease (ind. adm.), n (%)	1604 (4.0)	31 (2.8)	0.05

Chronic obstructive pulmonary disease, n (%)	1650 (4.1)	43 (3.9)	0.74
Chronic kidney disease, n (%)	1835 (4.6)	62 (5.6)	0.09
Chronic kidney diseases (ind. adm.), n (%)	1677 (4.2)	29 (2.6)	0.01
Other chronic disease (liver, pancreas, intestine), n (%)	522 (1.3)	7 (0.6)	0.05
Other chronic disease (liver, pancreas, intestine) (ind. adm.), n (%)	249 (0.6)	3 (0.3)	0.14
Previous coronary revascularization, n (%)	1835 (4.6)	60 (5.4)	0.17
Previous coronary revascularization (ind. Adm.), n (%)	802 (2.0)	25 (2.3)	0.52
Previous vascular surgery, n (%)	1649 (4.1)	40 (3.6)	0.43
Covid-19 diagnosis, n (%)	0	1102 (100)	<0.001
Thrombolysis<48 hours, n (%)	4917 (12.2)	102 (9.3)	0.003
Neurological unit, n (%)	28238 (70.2)	510 (46.3)	<0.001
LOS (mean ± SD)	9.5±7.7	13.9±12.6	<0.001

Abbreviations: Covid-19: coronavirus disease 2019; ind. adm.: index admission; LOS: length of stay

SUPPLEMENTARY TABLE S3. Baseline characteristics of the enlisted population by year.

	2015<br>N=54026	2016<br>N=53566	2017<br>N=52145	2018<br>N=50131	2019<br>N=49720	2020<br>N=41302	p value*
Gender (females), n (%)	27038 (50.0)	26705 (49.9)	26127 (50.1)	24617 (49.1)	24143 (48.6)	19924 (48.2)	0.61
Age (years), mean±SD	76.0±12.2	76.0±12.2	76.0±12.3	75.8±12.4	75.8±12.4	75.5±12.4	<0.001
Malignant neoplasms, n (%)	4394 (8.1)	4377 (8.2)	4123 (7.9)	4085 (8.1)	3938 (7.9)	3306 (8.0)	0.66
Diabetes mellitus, n (%)	6144 (11.4)	5871 (11.0)	5466 (10.5)	4997 (10.0)	4636 (9.3)	3579 (8.7)	0.24
Dislipidemias, n (%)	2218 (4.1)	2139 (4.0)	1952 (3.7)	1795 (3.6)	1693 (3.4)	1355 (3.3)	0.63
Obesity, n (%)	869 (1.6)	791 (1.5)	848 (1.6)	708 (1.4)	676 (1.4)	587 (1.4)	0.25
Obesity (ind. adm.), n (%)	572 (1.1)	549 (1.0)	448 (0.9)	542 (1.1)	537 (1.1)	612 (1.5)	<0.001
Anemia, n (%)	2625 (4.9)	2574 (4.8)	2504 (4.8)	2390 (4.8)	2309 (4.6)	1747 (4.2)	0.005
Anemia (ind. adm.), n (%)	1283 (2.4)	1281 (2.4)	1278 (2.5)	1206 (2.4)	1169 (2.4)	1021 (2.5)	0.42
Blood clotting defects, n (%)	142 (0.3)	120 (0.2)	94 (0.2)	91 (0.2)	87 (0.2)	69 (0.2)	0.31
Blood clotting defects (ind. adm.), n (%)	62 (0.1)	61 (0.1)	64 (0.1)	53 (0.1)	48 (0.1)	48 (0.1)	0.42
Other hematological diseases, n (%)	334 (0.6)	327 (0.6)	318 (0.6)	284 (0.6)	247 (0.5)	241 (0.6)	0.08
Other hematological diseases (ind. adm.), n (%)	239 (0.4)	236 (0.4)	239 (0.5)	250 (0.5)	233 (0.5)	227 (0.5)	0.28
Hypertension, n (%)	11703 (21.7)	10952 (20.4)	10147 (19.5)	9033 (18.0)	8412 (16.9)	6430 (15.6)	0.53
Previous myocardial infarction, n (%)	2838 (5.3)	2715 (5.1)	2559 (4.9)	2326 (4.6)	2246 (4.5)	1727 (4.2)	0.37
Heart failure, n (%)	4794 (8.9)	4648 (8.7)	4445 (8.5)	4088 (8.2)	3928 (7.9)	2958 (7.2)	0.004
Chronic coronary syndromes, n (%)	5274 (9.8)	5076 (9.5)	4805 (9.2)	4236 (8.4)	4125 (8.3)	3109 (7.5)	0,08
Rheumatic heart disease, n (%)	732 (1.4)	678 (1.3)	676 (1.3)	594 (1.2)	586 (1.2)	406 (1.0)	0,04
Rheumatic heart disease (ind. adm.), n (%)	388 (0.7)	371 (0.7)	376 (0.7)	385 (0.8)	401 (0.8)	421 (1.0)	0,002
Cardiomyopathy, n (%)	884 (1.6)	764 (1.4)	782 (1.5)	724 (1.4)	615 (1.2)	510 (1.2)	0.79
Cardiomyopathy (ind. adm.), n (%)	302 (0.6)	287 (0.5)	245 (0.5)	217 (0.4)	196 (0.4)	195 (0.5)	0,006
Endocarditis and acute myocarditis, n (%)	53 (0.1)	87 (0.2)	72 (0.1)	69 (0.1)	71 (0.1)	52 (0.1)	0.26
Arrhythmias, n (%)	7271 (13.5)	6936 (12.9)	6481 (12.4)	5993 (12.0)	5615 (11.3)	4271 (10.3)	0.02
Other chronic heart conditions, n (%)	1349 (2.5)	1309 (2.4)	1337 (2.6)	1212 (2.4)	1148 (2.3)	977 (2.4)	0,71
Other chronic heart conditions (ind.adm.), n (%)	1430 (2.6)	1284 (2.4)	1277 (2.4)	1327 (2.6)	1273 (2.6)	1257 (3.0)	<0.001
Vascular disease, n (%)	2875 (5.3)	2750 (5.1)	2513 (4.8)	2339 (4.7)	2229 (4.5)	1692 (4.1)	0.29
Vascular disease (ind. adm.), n (%)	1658 (3.1)	1764 (3.3)	1701 (3.3)	1745 (3.5)	1879 (3.8)	1635 (4.0)	0,46

Chronic obstructive pulmonary disease, n (%)	3475 (6.4)	3158 (5.9)	2898 (5.6)	2473 (4.9)	2378 (4.8)	1693 (4.1)	0.30
Chronic kidney disease, n (%)	3043 (5.6)	2931 (5.5)	2907 (5.6)	2664 (5.3)	2497 (5.0)	1897 (4.6)	0.007
Chronic kidney diseases (ind. adm.), n (%)	2635 (4.9)	2369 (4.4)	2274 (4.4)	2205 (4.4)	2099 (4.2)	1706 (4.1)	0.59
Other chronic disease (liver, pancreas, intestine), n (%)	1034 (1.9)	975 (1.8)	861 (1.7)	779 (1.6)	752 (1.5)	529 (1.3)	0.27
Other chronic disease (liver, pancreas, intestine) (ind. adm.), n (%)	509 (0.9)	445 (0.8)	429 (0.8)	376 (0.8)	393 (0.8)	252 (0.6)	0.07
Previous coronary revascularization, n (%)	2588 (4.8)	2539 (4.7)	2551 (4.9)	2355 (4.7)	2366 (4.8)	1895 (4.6)	0.2
Previous coronary revascularization (ind. Adm.), n (%)	1231 (2.3)	1212 (2.3)	1183 (2.3)	1039 (2.1)	1034 (2.1)	827 (2.0)	0.89
Previous vascular surgery, n (%)	2348 (4.3)	2290 (4.3)	2197 (4.2)	2135 (4.3)	2136 (4.3)	1689 (4.1)	0.27
Covid-19 diagnosis	0	0	0	0	0	1102 (2.7)	<0.001
Thrombolysis<48h, n (%)	3643 (6.7)	4768 (8.9)	5467 (10.5)	5813 (11.6)	6181 (12.4)	5019 (12.2)	<0.001
Neurological unit, n (%)	30816 (57.0)	32203 (60.1)	32740 (62.8)	32092 (64.0)	33077 (66.5)	28748 (69.6)	<0.001
LOS (mean ± SD)	10.4±10.3	10.4±9.2	10.1±8.4	10.2±8.5	10.2±8.6	9.6±7.9	<0.001

* p_values refer to the comparison between the observed in 2020 and the expected based on the previous five years trend

Abbreviations: Covid-19: coronavirus disease 2019; ind. adm.: index admission; LOS: length of stay

SUPPLEMENTARY TABLE S4. Logistic regression model for 30-day mortality

	**OR**	**95% CI**	
Gender (females)	1.09	1.06	1.12
Age (years)	1.09	1.09	1.09
Pre-Covid-19	**Ref**		
Lockdown – No Covid-19	**1.46**	**1.35**	**1.58**
Lockdown - Covid-19	**3.68**	**2.75**	**4.88**
PostLockdown – No Covid-19	**1.16**	**1.11**	**1.20**
PostLockdown - Covid-19	**2.49**	**2.08**	**2.97**
Malignant neoplasms	1.20	1.15	1.25
Diabetes mellitus	1.25	1.21	1.30
Dislipidemias	0.76	0.71	0.81
Anemia	1.25	1.19	1.31
Anemia (ind. adm.)	0.81	0.75	0.87
Blood clotting defects	1.14	0.92	1.41
Blood clotting defects (ind. adm.)	2.27	1.56	3.23
Other hematological diseases	1.27	1.11	1.45
Hypertension	0.95	0.92	0.98
Previous myocardial infarction	1.11	1.04	1.17
Heart failure	1.50	1.44	1.56
Rheumatic heart disease	1.14	1.04	1.24
Rheumatic heart disease (ind. adm.)	0.59	0.51	0.69
Cardiomyopathy	1.15	1.06	1.26
Other chronic heart conditions	1.07	1.00	1.15
Other chronic heart conditions (ind.adm.)	0.76	0.68	0.84
Arrhythmias	1.32	1.28	1.37
Vascular disease	1.15	1.08	1.22
Vascular disease (ind. adm.)	0.53	0.48	0.57

Chronic obstructive pulmonary disease	1.22	1.17	1.28
Chronic kidney disease	1.25	1.20	1.31
Other chronic disease (liver, pancreas, intestine)	1.26	1.15	1.37
Previous coronary revascularization	0.82	0.77	0.88
Previous vascular surgery	1.34	1.26	1.42
Neurological department	0.60	0.59	0.62

Abbreviations: Covid-19: coronavirus disease 2019; ind. adm.: index admission

SUPPLEMENTARY FIGURE S1. Expected (exp.) and observed (obs.) mortality rate at 30 days (in all ischemic stroke patients and in those without Covid-19 infection) during the 2020 and over the equivalent periods in the previous 5 years in Italy and by geographic regions.

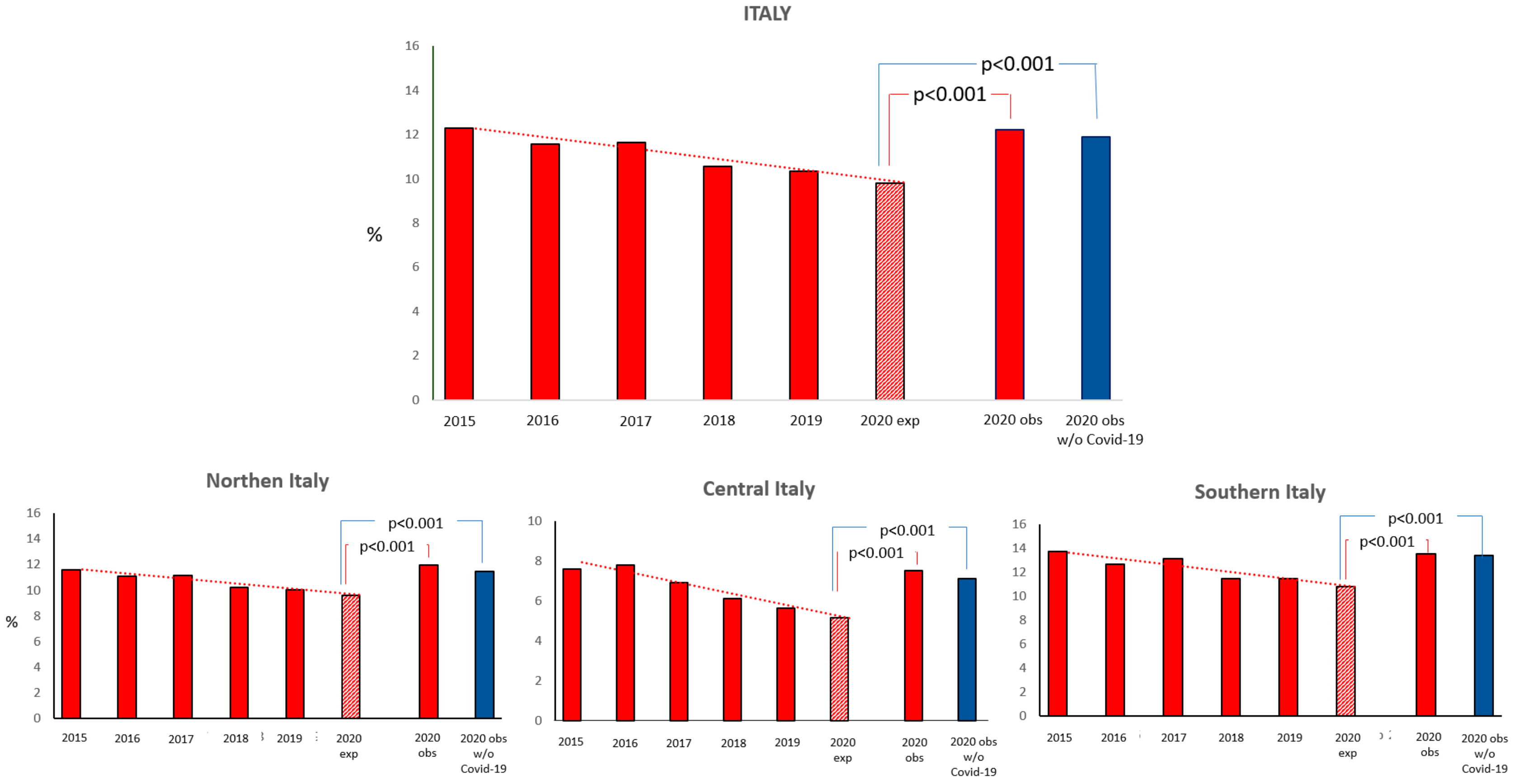

SUPPLEMENTARY FIGURE S2. Mortality rate at 30 days (panel A) and at 1 year (panel B) among patients without Covid-19 infection admitted in neurology and non-neurology units during the 2020 and over the equivalent periods in the previous 5 years in Italy.

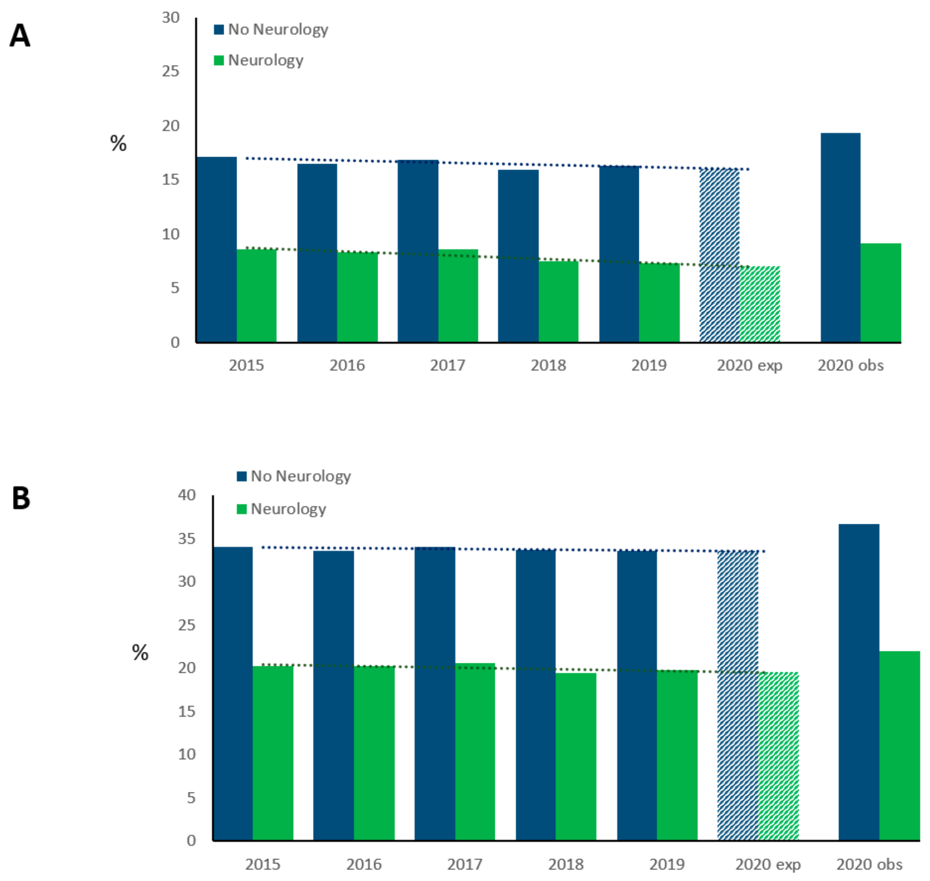

Supplement: Supplementary file 1 [file jcm-12-04560-s001.zip › jcm-2405879-SI.pdf]
